# Investigating socio-ecological factors influencing implementation of tuberculosis infection prevention and control in rural Papua New Guinea

**DOI:** 10.1093/pubmed/fdae018

**Published:** 2024-02-07

**Authors:** Gigil Marme, Jerzy Kuzma, Peta-Anne Zimmerman, Neil Harris, Shannon Rutherford

**Affiliations:** School of Medicine & Dentistry (Public Health), Griffith University, Gold Coast, QLD 4215, Australia; Department of Medicine, Divine Word University, Madang Province 511, Papua New Guinea; Graduate Infection Prevention and Control Program, School of Nursing and Midwifery, Griffith University, Gold Coast, QLD 4215, Australia; Higher Degree Research, Health Group, School of Medicine and Dentistry (Public Health), Griffith University, Gold Coast, QLD 4215, Australia; School of Medicine & Dentistry (Public Health), Griffith University, Gold Coast, QLD 4215, Australia

**Keywords:** health systems, Papua New Guinea, rural health, socio-ecological model, tuberculosis infection prevention and control

## Abstract

**Background:**

Tuberculosis (TB) is a highly transmissible infectious disease killing millions of people yearly, particularly in low-income countries. TB is most likely to be transmitted in healthcare settings with poor infection control practices. Implementing TB infection prevention and control (TB-IPC) is pivotal to preventing TB transmission in healthcare settings. This study investigated diverse stakeholders’ perspectives relating to barriers and strategies for TB-IPC in rural hospitals in Papua New Guinea.

**Methods:**

Multiple qualitative case studies were conducted with 32 key stakeholders with experience in TB services. Data collection drew on three primary sources to triangulate data: semi-structured interviews, document reviews and field notes. The data were analyzed using hybrid deductive-inductive thematic analysis.

**Results:**

Our results reveal that key stakeholders perceive multiple interdependent factors that affect TB-IPC practice. The key emerging themes include strategic planning for and prioritizing TB-IPC guidelines; governance, leadership and accountability at the provincial level; community attitudes towards TB control; institutional capacity to deliver TB care, healthcare workers’ safety, and long-term partnership and integration of TB-IPC programmes into the broad IPC programme.

**Conclusions:**

The evidence suggests that a multi-perspective approach is crucial for TB-IPC guidelines in healthcare institutions. Interventions focusing on addressing health systems strengthening may improve the implementation of TB-IPC guidelines.

## Introduction

Tuberculosis (TB) is one of the leading global health challenges of the 21st century,^1^^,^^2^ affecting ~10.6 million people with more than 1.6 million TB deaths registered annually in 2021.^3^ TB transmission in clinical settings is a critical public health problem.^4–6^ The risk of TB transmission is high in healthcare institutions with poor infection prevention and control (IPC) measures.^5^^,^^7^^,^^8^ A review of the literature revealed that poor ventilation systems, inadequate isolation facilities and personal protective equipment such as respirators were responsible for the TB transmission in resource-poor healthcare settings.^9^ Healthcare institutions are also likely to host many individuals with undiagnosed active TB posing an increased risk of transmission for healthcare workers (HCWs), patients and the broader community.^4^^,^^7^^,^^10^ This establishes the significance of adequate IPC interventions in healthcare institutions.

TB-IPC measures to effectively minimize TB transmission in healthcare facilities are well documented.^5^^,^^11–13^ In low- and middle-income countries (LMICs), where TB incidence is high, implementing TB-IPC guidelines in healthcare settings is consistently reported to be poor.^9^ Healthcare system challenges, including health workforce, financing, drugs, infrastructure, leadership and governance, health services delivery and health policy, are familiar explanations for poor implementation.^14^ These challenges, combined with the inadequate implementation of TB-IPC policy, significantly stall TB prevention opportunities.

A significant health policy goal of the post-2015 global ‘End TB Strategy’ is to reduce TB mortality rates by 95% by 2035.^1^ This health goal is reflected in the ‘PNG Strategic Development Plan 2010–2030’, which aims to reduce TB incidence from 342/100 000 population in 2015 to 150/100 000 by 2030.^15^ A key strategy is the effective implementation of TB-IPC guidelines in healthcare settings.^16^ Effective TB-IPC practice depends on promptly identifying TB infections, use of transmission-based precautions, and adequately managing health institutions to prevent overcrowding and improve patient flow.^17^ Better TB-IPC could be achieved by addressing barriers that influence TB-IPC measure implementation in resource-constrained healthcare settings.

Numerous researchers have discussed barriers to effective TB-IPC implementation in these settings with the causes often associated with multiple socio-ecological factors.^18^^,^^19^ However, insufficient research on the socio-ecological determinants influencing TB-IPC measures in rural health settings, including in PNG, means that insufficient information is available to help decision-makers and policy implementers in these settings to invest in the range of options across the levels of the socio-ecological model (SEM).

This study employed the SEM to explore intrapersonal, interpersonal, institutional, community and public policy interdependencies (Table 1).^18^ The SEM provides the framework to explore the complex interplay between proximal and distal determinants including impact of public healthcare policy on community values, attitudes and behaviours towards TB-IPC.^18^ This framework has been used in public health research to assess implementation barriers, including tuberculosis control in migrant studies,^20^ diabetes education,^18^ a community-based drug treatment programme^21^ and a childhood immunization programme.^22^ This qualitative study uses the SEM to frame a multilevel analysis and to understand what interventions may mitigate barriers to implementing TB-IPC guidelines in rural hospitals.

**Table 1:** SEM of TB-IPC measures

Levels	Socio-ecological factors
**Public policy level**	Leadership and governance Healthcare system strengthening Health policy and strategic planning Healthcare resource allocation
**Community level**	Traditional values and beliefs Education and awareness Access to healthcare services Neighbourhoods
**Institutional level**	Healthcare infrastructure Staffing Medical equipment Availability of and access to health policy
**Interpersonal level**	Teamwork and collaboration Family members Peers Professional network
**Intrapersonal level**	Training and education Knowledge, skills and experiences Attitudes Motivation Exposure to health risk

Adapted from Peterson^25^.

## Methods

### Study design

An explanatory multiple qualitative case study design was chosen to gain an in-depth understanding of TB-IPC practices in PNG rural hospitals.^23^ Rural hospitals were selected for this study because they are a resource-poor healthcare setting in PNG. Despite this limitation, they provide comprehensive TB care to the rural majority. Furthermore, more than 85% of the population lives in rural districts with limited healthcare access and poor living conditions, making them vulnerable to infectious diseases including TB.^24^^,^^25^ Many healthcare organizations today operate in volatile socio-economic environments and qualitative research plays a pivotal role in exploring organizational and management challenges.^26^ This research used multiple data collection techniques from multiple sources. Our study builds on a prior survey on TB-IPC guidelines in 10 PNG rural hospitals,^16^ which identified the need to better understand the socio-ecological factors shaping TB-IPC implementation. Cases were purposefully selected by five researchers involved in IPC and public health practice. Case selection was based on four TB-IPC performance indicators using the World Health Organization (WHO) IPC Assessment Framework.^16^ Contrasting cases (lower versus higher performing health facilities) were selected to understand and explain contextual conditions that led to different implementation outcomes. Four cases were chosen from two neighbouring regions, Highlands and Momase, the most populous PNG regions.^27^

### Participants and recruitment

HCWs, patients and community members were purposively sampled from the selected four rural hospitals (cases). The participants were approached through emails and phone calls with support of the hospital administrators. To introduce the participants to the study, participant information and consent forms were emailed to the administrators of the chosen facilities to identify staff and patients with experience in TB care to invite their participation. Participants were verbally briefed before interviews. In addition, provincial health managers were approached through emails and phone calls and appointments made for their participation. They were interviewed confidentially at the participant’s nominated location and time at the hospital.

### Data collection

Data was collected between April and June 2022. Thirty-two key stakeholders were interviewed using a semi-structured interview guide underpinned by the research objectives and the SEM framework. To assess the validity of each question, a pilot interview was conducted in a non-participating facility with the guide adjusted accordingly. Before the interviews, the researcher explained the purpose of the study and provided participants with the opportunity to ask questions during the interviews. All stakeholders were interviewed by the principal investigator, a male PNG national, with training and experience in mixed-methods research in PNG. Each interview lasted ~30 min.

To improve research credibility, the data collection drew on three primary sources to triangulate data: semi-structured interviews, document reviews and field notes captured throughout the study.^28^ For each case, semi-structured interviews were audio-recorded and transcribed verbatim. Health services documents, including strategic plans, operational plans, health promotion posters and health services reports, were reviewed to generate a clear understanding of the policies and policy environment related to TB-IPC policy. In addition, field notes were taken throughout interviews to summarize the main points and to capture information emphasized by participants.^29^ Member checking of synthesized data was undertaken by emailing participants a summary of the results to confirm the results resonated with their views.^30^

### Data analysis

Theoretical saturation was reached at interview 16, where no new information of interest emerged. However, the researcher interviewed all available and willing participants (*n* = 32) to support the findings. All three forms of data (interviews, documents, field notes) were analyzed using a hybrid deductive-inductive approach.^31^ This study used pre-determined themes and categories from the SEM and the emerging themes from the fieldwork.^32^ Interviews were transcribed and coded using NVivo 12 qualitative data analysis software.^33^ Documents and field notes were coded manually. Thematic analysis was applied to identify, analyze, interpret and report themes within the dataset.^34^ The researchers followed the five steps of the thematic analysis proposed by Clarke and Braun.^34^ First, interview transcripts were read and re-read to gain familiarization with the data. Second, one researcher coded interview transcripts systematically across the entire data set. Third, the investigation team met regularly to review the codes and to identify, develop and review themes until agreement was reached.^35^ Finally, the lead researcher refined each theme and extracted quotes from the dataset to support each theme.

### Ethical considerations

Ethical approval was given by the Griffith University Human Research Ethics Committees, Australia (GU Ref No 2021/921) and PNG Medical Research Advisory Committee (MRAC #22.01), and local procedures and requirements were adhered to when accessing health facilities. Verbal and written informed consent was recorded for all participants before the start of all interviews. Participation in the study was voluntary, with participants free to withdraw from the study without consequences. They were also advised of their right to refuse to answer any questions.

## Results

**Fig. 1:**
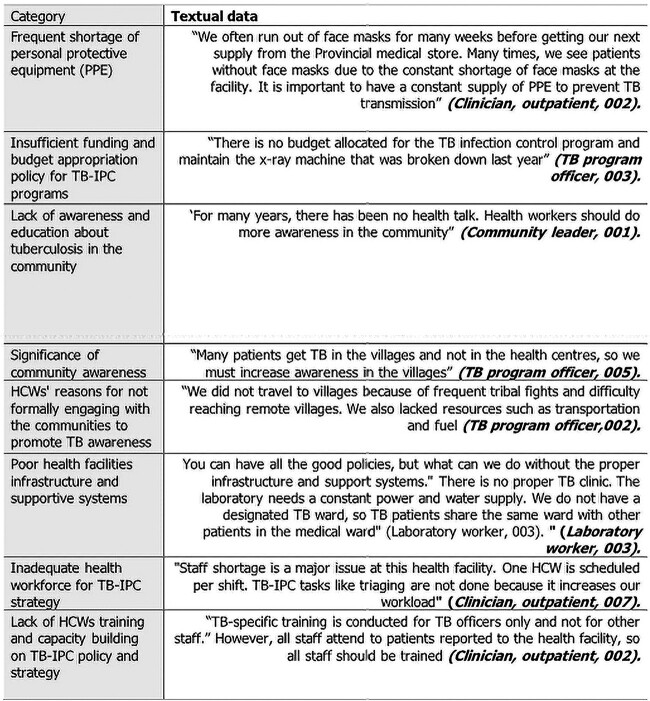
Excerpts from key informants’ interviews regarding healthcare institutions’ capacity to deliver TB control healthcare services.

In total, 32 HCWs (9 managers and policymakers, 13 clinical staff) and 10 patients (representing the community) were interviewed. There were 18 male and 14 female participants. The average age of the participants was 35 years. Many HCWs had been working in the field for over 10 years (*n* = 21/32). Six broad themes emerged from the data: strategic planning for and prioritizing TB-IPC policy; governance, leadership and accountability at the provincial health authority level; community behaviour, HCWs’ safety, and long-term partnership and integration of TB-IPC policy with the general IPC programme and multisectoral approach to managing TB-IPC programmes (Table 2). Healthcare institutions’ capacity to deliver TB care services forms a significant part of the emerging themes. The excerpts from the key informants are shown in Fig. 1. The emerging themes and sub-themes are discussed in the following text (Table 2).

**Table 2:** Breakdown of themes identified in this explanatory qualitative study

Themes	Sub-themes
Strategic planning for and prioritizing TB-IPC policy	Lack of recognition of TB-IPC policy in strategic planning at national and provincial level Absence of TB-IPC policies in provincial and health facilities implementation plans
Governance, leadership and accountability at the provincial and district level	Lack of supportive supervision, monitoring and mentoring from district and provincial health level Lack of leadership and accountability to approve funding for rural health services at the provincial health authority level
Community attitudes towards the delivery of TB control healthcare services	Patients delay in seeking healthcare due to the influence of traditional beliefs Stigma and discrimination amongst family members and relatives of TB patients
Capacity to deliver TB control healthcare services in rural health facilities	Frequent shortage of personal protective equipment in district hospitals Poor TB-IPC healthcare infrastructure and supportive systems in district hospitals Long-term shortage of key healthcare personnel in district hospitals Insufficient funding and budget appropriation for TB control healthcare services for district hospitals Lack of HCWs training and capacity building on TB-IPC policy and strategy Lack of TB and IPC health education and awareness in the community
Healthcare worker safety	HCWs have high workloads and are burnt out HCWs are exposed to TB due to frequent shortages of personal protective equipment
Long-term advocacy and integration of TB infection control policy with the general IPC programme	Insufficient participation and support from the three tiers of the health sector, development partners and non-government organizations Limited promotion of activities to communicate and advocate TB-IPC activities in healthcare institutions Partnership with district administration is important to harness the health programmes but is not sufficient

### Strategic planning for and prioritizing TB-IPC policy

#### Lack of recognition of TB-IPC policy in strategic planning at the national and provincial level

Although TB-IPC policy has been identified as one of the primary TB preventive measures in the National TB Management Plan, it was not identified as a priority health outcome in health plans. According to our document review, none of the TB-IPC guidelines were captured in the PNG health sector plans. The document review corroborates HCWs’ perceptions that the lack of strategic direction concerning TB-IPC policy is systemic. Participants said:

‘*TB infection prevention and control policy were introduced in the healthcare system since 2011 as an important TB prevention strategy and should be a priority strategy in all health plans.’ (TB Programme Manager, 003)*

### Lack of good leadership and accountability at the provincial health authority level

In conjunction with health services funding protocols, participants expressed the need for good management, leadership and accountability in approving funding for public health services at the Provincial Health Authority level. Participants stressed the importance of the timely approval and dispersal of public health funding:

‘*The delay in approving funding is an indication of lack of good management and leadership in the provincial health system.’ (TB Programme Officer, 003)*

### Community attitudes towards the delivery of TB control healthcare services

#### Patient delay in seeking healthcare due to the influence of traditional beliefs and values

Cultural beliefs appeared to influence patients’ understanding of TB. This included the firm belief in rural communities that the symptoms of the disease and related deaths are linked with sorcery, witchcraft and poison, not TB complications. One participant mentioned:

‘*Traditional belief is widespread in this village. When someone dies from TB, we think he/she has been bewitched.’ (Community leader, 002)*

In conjunction with these traditional beliefs, HCWs stated that traditional therapies often led to poor prognosis and health outcomes:

‘*Patients tend to seek traditional treatment and often are prescribed incorrect treatment. They only report to health centres when their condition deteriorates, often leading to deaths.’ (HCW, 004)*

#### Stigma and discrimination amongst family members and relatives of TB patients

TB was profoundly associated with stigma, and this has resulted in delays in seeking healthcare. HCWs agreed that the impact of stigmatization could be alleviated if HCWs deliver treatment to patients’ homes or if the community treatment supporter strategy through the Directly Observed Treatment Shortcourse (DOTS) is strengthened:

‘*Stigma with TB patients is common in the villages. Some families do not support the patient due to fear of being infected with the disease. Support the treatment supporter in the village to make medications available to patients at home.’ (HCW, 006)*

### Healthcare worker safety

#### Health workers have high workloads and are burnt out

All participating healthcare staff indicated the risk of being exposed to physical and mental health problems. The participants mentioned being overworked with limited compassionate leave and experiencing burnout. Participants relate the increasing workload and burnout in conjunction with the long-term shortage of healthcare staff at the health facility. A male nursing officer claimed:

‘*I work almost daily with limited days off due to staff shortage. I am tired and burnt out.’ (Clinician, 004)*

### Long-term partnership and integration of TB infection control policy with the general IPC programme

#### Intersectoral cooperation from the health sector, development partners and non-government organizations

Many interviewees regard intersectoral cooperation between the health sector, development partners and non-government organizations as the most important facilitator of good TB-IPC practices in healthcare organizations. Participants emphasized that working with key partners is essential to increase capacity and sustain the TB-IPC programme.

‘*TB is a complex health issue. We need support from the different tiers of the health sector and international partners to provide TB care.’ (Health facility manager, 005)*

Other respondents emphasized the importance of promoting specific activities to communicate and advocate TB-IPC activities in healthcare institutions. For example, they explained that TB-IPC activities such as using personal protective equipment (e.g. gloves, masks, gowns), aseptic procedures, hand hygiene and environmental control measures should be integrated with the general IPC plans to promote long-term sustainability of TB-IPC practice in healthcare:

‘*The TB infection control activities should be integrated with the IPC program to share resources. This is good for the long-term sustainability of the TB control program.’ (Provincial Health Manager, 003)*

## Discussion

### Main findings of this study

This study provides insights into key stakeholders’ perspectives on barriers and strategies of TB-IPC policy implementation in rural PNG. Overall, our results reveal that key stakeholders perceive multiple interdependent socio-ecological barriers that affect TB-IPC and require multi-component strategies to improve TB-IPC in rural PNG. The main perceived barriers fall into six broad and interconnected categories and as such our findings suggest the need for a multifaceted approach guided by the socio-ecological framework to address the shortfall in TB-IPC policy implementation.

Our findings are consistent with Zwama *et al*.^10^, who suggest that the successful implementation of TB-IPC depends on understanding the influences of the health system and the broader environment. TB can be complicated and difficult to manage from micro-level analyses and therefore requires a system thinking approach to address the complexities. This aligns with the findings of Mdegela *et al*.^36^, who adopted SEM as a theoretical framework to appraise health workforce retention in Malawi and Tanzania, asserting that a broader perspective may be needed to better understand both the risk and the intervention side of public health programmes.^37^^,^^38^ This multi-perspective research outlines barriers beyond the proximal determinants and highlights crucial upstream determinants that impede TB-IPC standards in rural hospitals.^39^ Managing these determinants is pivotal if PNG is to comply with the global campaign of the WHO to eradicate TB in all LMICs.

This study shows that strategic planning for and prioritizing TB-IPC policy is a significant TB control strategy in PNG. This finding is consistent with Dennis,^40^ who maintains that strategic planning is essential for any organization aiming to secure long-term success. It enhances decision-making, improves resource management, provides direction and increases operational efficiency. Whilst strategic planning such as the TB-IPC policy is something health managers and policymakers developed at the national level, the subsequent implementation of this plan has been left ‘on the shelf’—referenced on occasion but for most part neglected as evidenced in this research. TB is a complex public health problem and requires comprehensive support from the strategic level.^41^ This means capturing TB-IPC guidelines in strategic planning, allocating resources and enabling HCWs to coordinate healthcare resources towards implementing targeted TB-IPC interventions. The key to successful strategic planning and implementation is engaging everyone in the planning process and designing measures and implementation strategies that will allow HCWs to monitor the results regularly.^42^

This study revealed that limited governance and leadership at the provincial health level has affected implementation of TB-IPC policy. This finding is critical because commitment to good governance drives successful services and organizations. This corroborates the findings of Cleary *et al*.^43^, who revealed that vibrant management and leadership competencies are essential for strengthening health systems. Similarly, accountability is pivotal in leadership as it promotes teamwork and coordination of activities towards achieving a common goal, including effective management of TB. This study not only highlights opportunities for stronger leadership but also identifies the consequences of leader–worker relationships in the healthcare facilities to maintain cohesion amongst HCWs for better healthcare services. Scott *et al*.^44^ emphasize that leader–worker relationships can lead to a lack of trust and respect between the leader and the team. However, when leaders are held accountable for their actions and understand the impacts of failing to meet expectations, employees will trust the leadership more, leading to improved performance. Thus, accountability can also accelerate innovation amongst HCWs, which can lead to better TB-IPC policy implementation outcomes in the long term.

The most important result was that healthcare institutions in the districts could not roll out TB-IPC programmes. Our findings reveal that although institutional capacities are significant for enabling effective policy implementation and healthcare services, this needs to be stronger in most rural health facilities for many reasons, including long-term shortage of HCWs, lack of medical technologies and drugs, inadequate funding for TB-IPC programme and poor healthcare infrastructure. This also accords with our earlier evaluation of TB-IPC policy implementation in rural districts, which showed strengthening healthcare systems is significant for providing functional and effective TB care services to those in need.^16^ These results reflect those of Oronje *et al*.^45^, who found that investment in health systems strengthening interventions can help improve and sustain health outcomes. Sustaining healthcare institutions’ capacity is complex and requires sustained leadership, management support, and long-term planning and investments to implement key public health policies. Although strong institutional capacity is critical for health policy implementation, this needs to be stronger in many LMICs, including PNG.^45^

From the perspective of the key stakeholders who participated in this study, TB is considered a complex public health issue involving the broader society. Therefore, developing partnerships between the national health sector and relevant agencies, including development partners, non-government organizations and Christian Health Services, is critical to strengthen the long-term implementation of TB-IPC policy to monitor the TB epidemic in PNG. This is consistent with WHO’s assertion of the significance of collaboration between multiple sectors towards TB prevention, care and control.^46^ This implies that the delivery of national TB programmes should be coordinated between organizations drawn from different sectors of society who are dedicated to working collaboratively towards TB control efforts. Whilst the government is responsible for delivering TB healthcare to patients, different actors can become active partners in TB prevention, care and control. At the upstream policy level, the health sector should engage other key government sectors, including housing, water, energy and businesses, and invest in improving housing, water and sanitation, electricity and income earning opportunities as a means to empower the most vulnerable populations.^47^

### What is already known

Active TB continues to remain in many countries, particularly in LMICs, primarily because of the ongoing reactivation of latent TB infection. As such, developing clear public health guidelines such as infection control practices can minimize or eradicate the presence of suspected or known TB cases in high TB burden healthcare settings.^48^ TB-IPC guidelines consist of evidence-based interventions intended to prevent individuals from being exposed to infectious TB and minimize the threat of transmitting infectious diseases. The most effective means of preventing transmission of TB is early diagnosis and initiating correct medications. Unfortunately, complex barriers of TB-IPC practices are commonly reported in resource-constrained healthcare settings in LMICs.^2^^,^^49^ Considering these complexities, a holistic perspective of the barriers to TB-IPC guidelines is necessary for improving individual (HCWs and patients), community, healthcare institutions and public policy level.

### What this study adds

The findings provide valuable information for policymakers and health managers to make informed decisions considering the complexity of the context, which drives TB-IPC policy implementation.^2^ Cross-cutting the health systems issues, key stakeholders identified the lack of priority accorded to TB-IPC in its implementation plans as a systemic barrier. Similar to other studies, TB-IPC should be prioritized in the health plans and supported with sufficient resources.^50^ This study supports the position that TB-IPC policy is not immune to the health system challenges driving implementation in resource-constrained health settings.^10^ Adopting a socio-ecological approach can identify systemic barriers across the different tiers of the health system that can inform the design of interventions to reduce the risk of TB transmission.^11^ Community involvement could foster a shift in the paradigm of care from a setting-based to a decentralized, population-based mode of healthcare. TB and IPC training should be offered to all HCWs, not just TB programme managers, to increase TB specialization human resources at the facility. Lack of HCWs training in TB prevention may influence compliance, as found in other studies conducted in Nigeria and China.^51^^,^^52^

### Limitations

There are several limitations in the study. First, the barriers identified in this research may differ in settings with different healthcare systems, community and healthcare institutions. Second, common to case study research, our study was context specific, and the results cannot be automatically generalized to other settings. However, several approaches were used in this study: the triangulation method, analyzing the code pattern and information saturation, and employing ongoing discussions about the obtained data amongst the research team. Therefore, such approaches contribute to the validity of our study.

## Conclusion

This study has presented key TB-IPC stakeholders’ perspectives on the barriers to and strategies for implementing TB-IPC guidelines in rural hospitals in PNG. The key findings suggest that stakeholders perceive that reducing TB incidence require concerted efforts from other sectors working with the health sector and adopting the socio-ecological approach to address the diverse levels of the healthcare system and implementing specific TB-IPC measures in healthcare settings. Interventions focusing on strengthening health systems, including improving healthcare settings capacity and HCW training, along with prioritizing TB-IPC policy, may improve implementation. Such initiatives should be taken by the national and provincial, and facility administrators.

## Data Availability

The data underlying this article are available in the article and in its online supplementary material.

## Notes

**Gigil Marme,** Lecturer, PhD Candidate

**Jerzy Kuzma,** Head of Department

**Peta-Anne Zimmerman,** Director, Graduate Infection Prevention and Control Program

**Neil Harris,** Director, Higher Degree Research, Health Group

**Shannon Rutherford,** Public Health Team
